# Complete Genome Sequence of the WHO International Standard for Hepatitis B Virus DNA

**DOI:** 10.1128/genomeA.01576-16

**Published:** 2017-02-16

**Authors:** Adrian Jenkins, Rehan Minhas, Clare Morris, Neil Berry

**Affiliations:** Division of Virology, National Institute for Biological Standards and Control, South Mimms, United Kingdom

## Abstract

The World Health Organization (WHO) international standard (IS) for hepatitis B virus (HBV) DNA for use in nucleic acid amplification assays was characterized by determining the complete genome sequence, which was assigned genotype A. This information will aid the design, development, and evaluation of HBV DNA amplification assays.

## GENOME ANNOUNCEMENT

Hepatitis B virus (HBV) remains a major public health problem worldwide. An estimated 350 million people worldwide have chronic HBV infection of which approximately 600,000 people die annually either from acute infection or cirrhosis and hepatocellular carcinoma caused by chronic infection (1). Direct amplification of the HBV DNA genome is routinely used in the clinical management of HBV infections, particularly to guide the initiation of and monitor the response to antiviral therapy in chronically infected patients (2). The HBV international standard is also used by in vitro diagnostics (IVD) manufacturers, blood transfusion centers, control authorities, and clinical laboratories to calibrate secondary reference materials and in the validation of HBV nucleic acid testing (NAT) assays (3). The candidate replacement WHO international standard (IS) for HBV DNA (designated 10/266), is to be evaluated to replace the current established HBV IS 10/264 in use since 2014 (3). The genetic composition of the HBV IS has not been described previously; hence, we report the complete genome sequence using Sanger sequencing.

The HBV IS was prepared from a dilution of the Eurohep reference R1 (genotype A2, HBsAg subtype adw2) virus stock held at NIBSC and also used for all previous standards. Following viral DNA extraction (Qiagen), PCR-amplification reactions were performed with HotStarTaqTM master mix (Qiagen, Hilden, Germany). Four independent sets of amplification and sequencing primers were applied as previously described by Chook et al. (4). Combinations were as follows with numbering according to HBV (strain ayw), NCBI reference sequence NC_003977.2: Set 1a: 251f GACTYGTGGTGGACTTCTC, 1190r TCAGCAAAYACTYGGCA; set 1b: 595f CACHTGTATTCCCATCCCA, 1797r CCAATTTMTGCYTACAGCCTC, 1190f AYGCAACCCCCACTGG; set 2a: 2300f CCACMWAATGCCCCTATC, 215r AGRAAMACMCCGCCTGT; set 2b: 2819f ACCWTATWCYTGGGAACAA, 617rc GAYGAYGGGATGGGAATACA, 654r GSCCCAMBCCCATAGG; set 3: 1859f ACTNTTCAAGCCTCCRAGCTG, 1877f CTGTGCCTTGGRTGGCTT, 2835r GTTCCCAVGWATAWGGTGAYCC; set 4: 1584f ACTTCGMBTCACCTCTGCACGT, 2331r GGAAGYGTKGAYARGATAGGGGCATT, 2396r GTCKGCGAGGYGAGGGAGTT.

After visualization on a 1% agarose gel, each amplicon was diluted in water before being used as a template for sequencing using a combination of PCR primers and suitable internal sequencing primers. Each sequencing reaction contained 4-μl cleaned PCR product, 0.5 μm primer, 3 μl reaction mix (ABI PRISM big dye terminator kit, Applied Biosystems) amplified using the cycling profile of 25 cycles of 96°C 30 s; 50°C 15 s; 60°C 4 min as recommended by the manufacturer’s protocol. Amplified products were precipitated with ethanol prior to running on an ABI 3130XL sequencer in accordance with the manufacturer’s instructions. Sequence was generated in both directions, assembled and analyzed using Geneious v7 software, and a consensus sequence generated.

This is the first report of the complete genome sequence of the WHO HBV DNA IS. The standard is used in development and evaluation of genome amplification assays for HBV DNA quantification, which provide important clinical data for management of HBV-infected patients. The complete consensus genome sequence reported here, derived from a viral DNA template, will support genome amplification assay development used in the clinical management of HBV-infected individuals and molecular epidemiological studies.

### Accession number(s).

The complete genome sequence of the WHO IS for HBV DNA reported here as a consensus has been deposited in GenBank under the accession number KY003230.
